# Dynamic Response of Elastomer-Based Liquid-Filled Variable Focus Lens

**DOI:** 10.3390/s19214624

**Published:** 2019-10-24

**Authors:** Lihui Wang, Masatoshi Ishikawa

**Affiliations:** 1 Guangdong Institute of Semiconductor Industrial Technology, Guangdong Academy of Sciences, No.363 Changxing Rd., Tianhe, Guangzhou 510-650, China; 2 Department of Information Physics and Computing, University of Tokyo, 7-3-1 Hongo, Bunkyo-ku, Tokyo 113-8656, Japan; masatoshi_ishikawa@ipc.i.u-tokyo.ac.jp; 3 Department of Creative Informatics, University of Tokyo, 7-3-1 Hongo, Bunkyo-ku, Tokyo 113-8656, Japan

**Keywords:** liquid-filled variable focus lens, aperture sizes, dynamic response, natural frequency

## Abstract

Variable focus lenses are capable of dynamically varying their focal lengths. The focal length is varied by adjusting the curvature of the refractive surface and the media on both sides of the lens. The dynamic response is one of the most important criteria to determine the performance of variable focus lens. In this work, we investigated critical factors that affect the dynamic response of liquid-filled variable focus lens with a large aperture size. Based on a theoretical analysis of a circular disk representative of a deformable surface, we found that the dynamic response is significantly influenced by the diameter, thickness, and stiffness of the disk because these factors determine its first natural frequency. We also studied the dynamic response of elastomer-based liquid-filled variable focus lens prototype with different aperture sizes (20 and 30 mm) by using experiments and we found that the lens with the smaller aperture size had an excellent dynamic response.

## 1. Introduction

In an optical system, the conventional method used to adjust the focal length of the system or zoom into an object is to employ a multitude of lenses and mechanically move these lenses over specific distances along the optical axis. Because the lenses are made of glass or plastic, the lenses have fixed optical properties after fabrication. Solid lenses can be processed to form a spherical or aspheric surface. Various materials are used to fabricate the lenses to fulfill the optical requirements of a specific application such as achromatic lenses made of crown and flint glass doublets. Specific materials such as silica glass and chalcogenide glass are also used to fabricate lenses for other spectrum imaging applications. Because of the fixed focal length of solid lenses, the response times of the focus and zoom functions are limited by the mechanical motion of the lenses in the optical system. The motors in the optical system move back and forth and the continuous forward and reverse rotations result in high power consumption. Hence, the functions of the optical system will be temporarily disabled by the built-in safety mechanism to prevent overheating caused by the frequent conversion of the positive and negative driving current. Such a mechanism is not suitable to achieve a high-speed dynamic response. 

A variable focus lens can dynamically change its focal length and therefore, only a single lens is needed in the optical system. Because a variable focus lens system does not require the back and forth motion of a multitude of lenses, variable focus lenses are favorable alternatives for optical design. Several compact optical systems with variable focus lenses have been reported [1,2,3,4,5,6,7,8,9,10,11]. Variable focus lenses can be used for visible, infrared, and terahertz (THz) imaging technologies based on the properties of the liquid candidates [12,13,14]. The focal length of a liquid-filled variable focus lens is varied by adjusting its bending curvature of the refractive surface, resulting in a rapid response time. Oku et al. [15] produced a silica glass-sealed tunable lens with a 1-kHz bandwidth frequency. Oku and Ishikawa [16] fabricated a lens prototype with a liquid-liquid interface. The prototype was driven by a piezo actuator and the maximum response time achieved was 2 ms. Maffli et al. [6] developed a dielectric elastomer-based tunable lens with a settling time of 0.175 ms. To achieve high optical performance for liquid-filled variable focus lens, it is crucial to ensure that the aperture size is small (i.e., diameter less than 10 mm) compared with the capillary length, while considering the physical constraints of the optical system [17]. If a liquid-filled variable focus lens has a large aperture size and the lens is positioned vertically, the lens profile may be asymmetrically deformed due to gravitational effects [18]. We [19] developed a variable focus lens with a liquid-membrane-liquid structure and large aperture size (a diameter of up to 30 mm). The lens was designed such that elastic force was the dominant force instead of surface tension force to significantly increase the capillary length. However, the dynamic response of this lens is not known. 

The dynamic response is one of the most important criteria for a variable focus lens and it is crucial to minimize the response time (settling time) of the lens. Hence, in this study, we investigated the critical factors that affect the dynamic response of an elastomer-based liquid-filled variable focus lens to minimize the response time. We studied the dynamic response of a circular disk model representative of a deformable surface (deformable plate and elastomer). We hypothesized that the diameter, thickness, and stiffness of the circular disk are the critical factors that influence the dynamic response of the liquid-filled variable focus lens. We fabricated the elastomer-based liquid-filled variable focus lens prototype with different aperture sizes (diameter: 20 and 30 mm) and we used a high-speed piezo stack actuator to measure the dynamic response of the lens with a change in the focal length. The piezo stack actuator was pushed into the deformable plate, whose diameter was larger than the aperture of the lens. The curvature of the variable focus lens formed by the transparent elastomer was passively controlled. 

Based on the experimental results, the lens prototype with an aperture size of 30 mm achieved a stable response at 30 Hz when we applied a sinusoidal wave as the active signal. The lens prototype with an aperture size of 20 mm achieved a stable response at 45 Hz. When we used a high-frequency sinusoidal wave, we observed that the amplitude of the feedback signal from the photodiode module significantly decreased, indicating that the vibrations of the elastomer surface were damped. When we applied a square wave as the active signal, we found that the settling times were 200 and 160 ms, respectively, when the lens prototype with an aperture size of 30 mm was switched from the relaxing phase to the contracting phase and vice versa. When the aperture size was reduced to 20 mm, the settling times of the lens prototype were significantly reduced to 60 and 40 ms, respectively, and the lens prototype was switched from the relaxing phase to the contracting phase and vice versa, indicating a marked improvement in the dynamic response of the lens prototype with a smaller aperture size. 

Our experimental results indeed indicate that the dynamic response of the liquid-filled variable focus lens is influenced by the diameter, thickness, and stiffness of the elastomer. Because our lens prototype can steadily vibrate to vary its focal length at a frequency of more than 30 Hz, the lens prototype can be potentially embedded in a high-speed projector to generate three-dimensional interactive scenes [20,21]. We believe that the lens prototype can also be used to realize a high-speed shape from focus and all-in-focus imaging [22,23]. Even though the elastomer used in our experiments can be easily fabricated, it lacks in terms of stiffness. If an elastomer with higher stiffness can be employed, this will considerably improve the dynamic response of the elastomer-based liquid-filled variable focus lens. Moreover, the dimensions of the lens can be tailored to fulfill the requirements of a particular application. We believe that the critical factors that affect the dynamic response of the liquid-filled variable focus lens identified in this work will be useful in the design of variable focus lens for optical systems. 

The manuscript is organized as follows. The design principle of the evaluation system is described in the Section 2. In Section 3, the building of the mathematical model of the system, the analysis of the dynamic performance of the deformable plate, and the building of the prototype is described. The experimental setups and procedures of the study are illustrated in this section. The results of the measurements and the discussions are presented in Section 4. The conclusions of the study are presented in Section 5.

## 2. Design Principle

**Figure 1:**
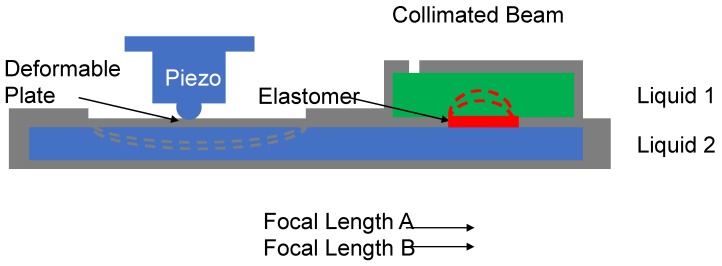
Schematic of the variable focus lens system (cross-sectional view). The liquid chambers are filled with different types of liquid (Liquid 1 and Liquid 2) and a piece of transparent elastomer is inserted in between to separate the liquids. The lower chamber is equipped with a deformable plate, which is passively deformed by a piezo stack actuator. The circular boundary of the elastomer is fixed such that the elastomer surface will change from a convex profile to a concave profile.

In this work, we focused on investigating the dynamic response of an elastomer-based variable focus lens with a large aperture size and therefore, we will not discuss variable focus lenses with a liquid-liquid structure. Our study is primarily focused on liquid-membrane and liquid-membrane-liquid variable focus lenses. Figure 1 shows the schematic of the elastomer-based variable focus lens system. In this design, two chambers are filled with different types of liquid and these liquid-filled chambers are separated by a piece of transparent elastomer. Because the liquids have different refractive indices, the curvature of the elastomer at the liquid-membrane-liquid interface can be considered as the lens surface. The liquids are assumed to be incompressible. 

The lower chamber (blue chamber) is equipped with a deformable plate such that the chamber volume is varied by the motion of the piezo stack actuator. When the piezo stack actuator is pushed downwards on the deformable plate, the volume of the lower chamber decreases and the elastomer becomes deformed. The elastomer is prepared by applying a homogeneous in-plane pretension force and its circular boundary is fixed such that the deformation profile of the elastomer is reminiscent of a paraboloid profile. Thus, the focal length changes due to changes in the bending curvature.

To monitor the high-speed variation of the elastomer surface, it is necessary to use a high-speed piezo stack actuator instead of a syringe pump. Even though the piezo stack actuator can realize displacement variations with a frequency in the order of kilohertz (kHz), the stroke length can only be one-thousandth of its body length. However, the motion of the piezo stack actuator is still too short to realize a considerable change in the liquid volume. For this reason, a built-in amplifying mechanism was designed [15]. The designed deformable area of the piezo stack actuator consists of two areas: (1) active deformable area with size S and (2) passive deformable area of the elastomer (i.e., the variable focus lens profile) with size s. Therefore, the lens surface will change by approximately S/s times relative to the deformable plate. With this design, we can realize a large elastomer deformation of the elastomer with only a small stroke length of the piezo stack actuator.

In this lens design, the deformable plate and elastomer were actively and passively vibrated, respectively. The natural frequencies of these components determined the dynamic response of the liquid-filled variable focus lens. Hence, we developed a mathematical model of the deformable plate and elastomer. We performed numerical simulations to determine their natural frequencies and select the optimal candidates for the deformable plate and elastomer.

## 3. Methodology

### 3.1. Mathematical Model

The elastomer-based liquid-filled variable focus lens consists of two parts that will vibrate: (1) a deformable plate and (2) an elastomer. Both the deformable plate and elastomer are circular disks with a fixed boundary, and therefore, they can be represented by the same mathematical model. It is essential for the deformable plate and elastomer to have a high-speed dynamic response and high natural frequency. 

The deformable plate is deformed by the piezo stack actuator and the elastomer profile is passively controlled. The elastomer profile is representative of a refractive lens surface and therefore, it is imperative to maximize the optical performance of the elastomer. 

The conventional method used to increase the natural frequency of a component is to build a tough structure and select a stiff material. However, the ability to deform is an essential criterion for a variable focus lens. Hence, the best candidates for the deformable plate and elastomer are materials that will maintain a high natural frequency and elasticity for deformation to take place. We chose the materials that would give the best trade-off in terms of the natural frequency and stiffness for the deformable plate and elastomer. 

**Figure 2:**
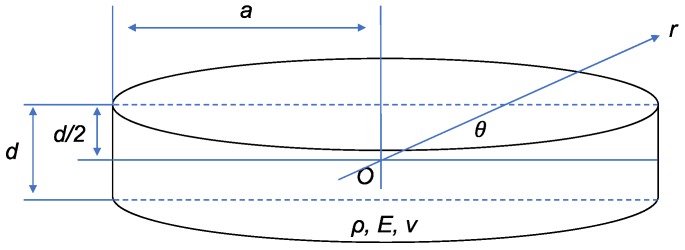
Schematic of the circular disk with a fixed boundary.

We developed the mathematical model to analyze the dynamic performance of the deformable plate and elastomer. Both these components have the same shape, and thus, they can be represented by the same mathematical model. Figure 2 shows the schematic of the circular disk with a fixed boundary, which is representative of the deformable plate or elastomer. The radius, thickness, Young’s modulus, density, and Poisson’s ratio of the circular disk are denoted by a, d, E, ρ, and v, respectively.

Based on the natural modes of vibration [24], the natural frequency w of the circular disk can be written as

(1) $$\omega_{m,n}=k_{m,n}^{2}d\sqrt{\frac{1}{12\left(1-v^{2}\right)}\frac{E}{\rho },}\;\left(m,n=0,1,2,\ldots \right),$$

where m and n denote the vibration modes. The parameter $k_{m,n}$ is the solution of

(2) $$I_{m}\left(k_{m,n}a\right)J_{m-1}\left(k_{m,n}a\right)-J_{m}\left(k_{m,n}a\right)I_{m-1}\left(k_{m,n}a\right)=0.$$

where $I_{m}$ is the mth modified Bessel function of the first kind and $J_{m}$ is the mth Bessel function of the first kind. A vibration mode where m=0 and n=1 corresponds to the first natural vibration. Note that $k_{m,n}$ is proportional to 1/a because it is the solution of Equation (2). Hence, it can be deduced that the first natural frequency $\omega_{0,1}$ is positively correlated to $1/a^{2}$, d, and $\sqrt{\frac{1}{\left(1-v^{2}\right)}\frac{E}{\rho }}$.

### 3.2. Dynamic Performance of the Deformable Plate

**Figure 3:**
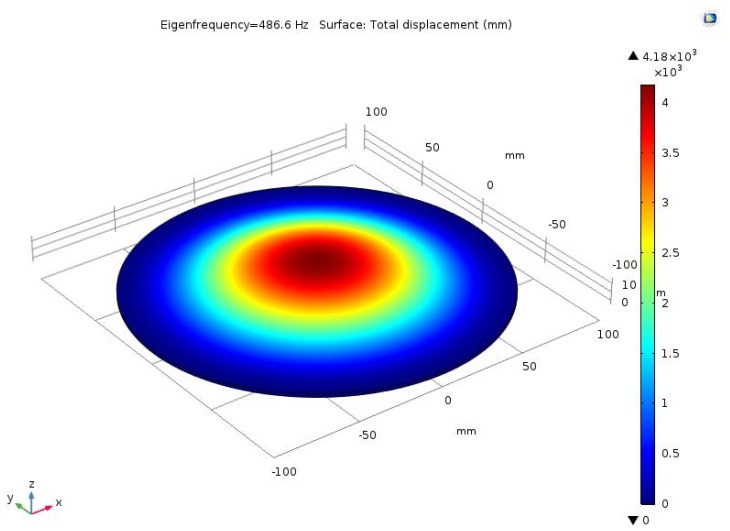
First natural frequency (eigenfrequency) of the rigid circular disk obtained from finite element simulations. The Young’s modulus, Poisson’s ratio, and density were set as ~192.9 MPa, ~0.3, and ~7930 kg/m^3^, respectively, which are representative of those for SUS304 stainless steel. The first eigenfrequency was found to be 486.63 Hz.

We performed finite element simulations to investigate the dynamic performance of the circular disk. We used COMSOL Multiphysics^®^ modeling software (Version 5.3 COMSOL Inc., Stockholm, Sweden) for this purpose, where the diameter 2a and thickness d of the rigid circular disk were set as 200 mm and 2 mm, respectively. The material properties of the deformable plate were set as follows: (1) Young’s modulus, E: ~192.9 MPa, (2) Poisson ratio, v: ~0.3, and (3) density, ρ: ~7930 kg/m^3^. These properties are representative of those for SUS304 stainless steel. Figure 3 shows the simulation result and the first eigenfrequency of the rigid circular disk was found to be 486.63 Hz. Because the deformable plate corresponds to the motion of the high-speed piezo stack actuator, the feedback response of the deformable plate should be less than 30% of its first eigenfrequency. Thus, we assumed that the most suitable dynamic vibrational frequency of the deformable plate was 146 Hz.

### 3.3. Fabrication of the Elastomer-Based Liquid-Filled Variable Focus Lens Prototype

**Figure 4:**
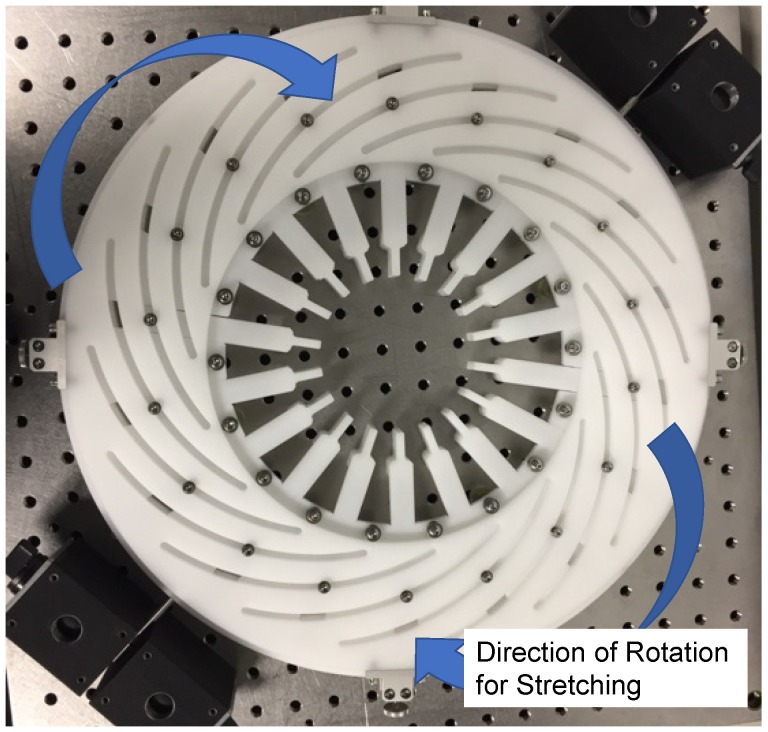
Photograph of the customized in-plane pretension force device. A piece of elastomer is placed at the center of the device and the elastomer is stretched over a certain period.

Stiffness is an important factor that will determine the first natural frequency of a component. The elastomer was too soft compared with the SUS304 stainless steel deformable plate. Hence, we implanted an in-plane pretension force device in the elastomer to increase its stiffness [17]. Figure 4 shows the customized pretension force device fabricated in this work. The pretension force device was fixed on top of a table and a piece of elastomer was fixed at the center of the device. With this device, the elastomer can be stretched, producing a homogeneous in-plane pretension force. The in-plane pretension force improves the optical performance of the variable focus lens, especially when the lens is positioned vertically [17].

**Figure 5:**
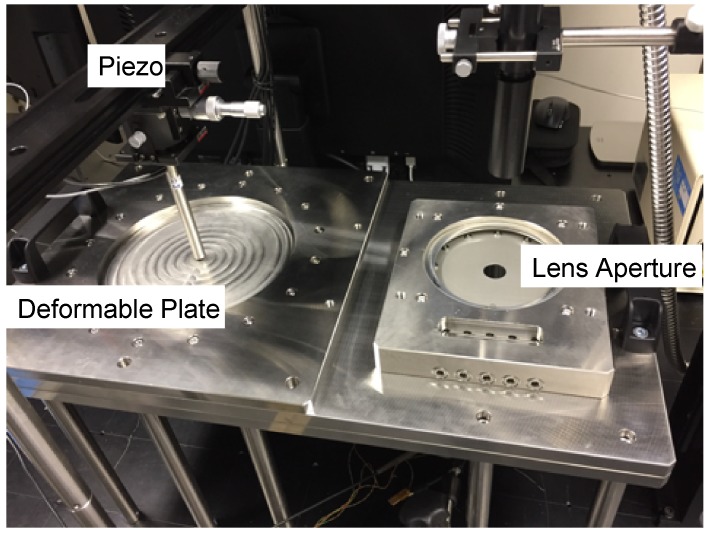
Photograph of the in-house experimental setup used to measure the dynamic response of the elastomer-based liquid-filled variable focus lens prototype with different aperture sizes.

Figure 5 shows the experimental setup fabricated in-house to measure the dynamic response of the elastomer-based liquid-filled variable focus lens prototype with different aperture sizes. The deformable plate had a diameter and thickness of 200 mm and 2 mm, respectively, as shown on the left-hand side of the photograph. A piezo stack actuator (Model: P-841.60B, Physik Instrumente (PI) GmbH & Co. KG, Germany) with a stroke length of 90 μm was used in this work, which was mounted on top of a sturdy platform and directed towards the center of the deformable plate. The liquid chambers were filled with different types of liquid (each with a different refractive index). The liquid chambers were connected to each other via a circular hole and a piece of elastomer was inserted in between to separate the two liquids. Polyacrylate (3M^TM^ VHB^TM^ Tape 4910, 3M, St. Paul, MN, USA) was chosen as the elastomer in this study [8]. The polyacrylate was originally designed as a double-sided tape and therefore, the polyacrylate elastomer was carefully taped to a doughnut-shaped plate. The elastomer was stretched to 1.5 times its original length to increase its natural frequency and following this, the elastomer was affixed to the plate. The plate was mounted and fixed to the circular hole between the two liquid chambers. The inner aperture serves as the circular boundary condition of the variable focus lens profile. We fabricated two plates with different aperture sizes (20 and 30 mm) to evaluate the dynamic response of the elastomer-based liquid-filled variable focus lens prototype with different aperture sizes.

### 3.4. Experimental Setup and Procedure

**Figure 6:**
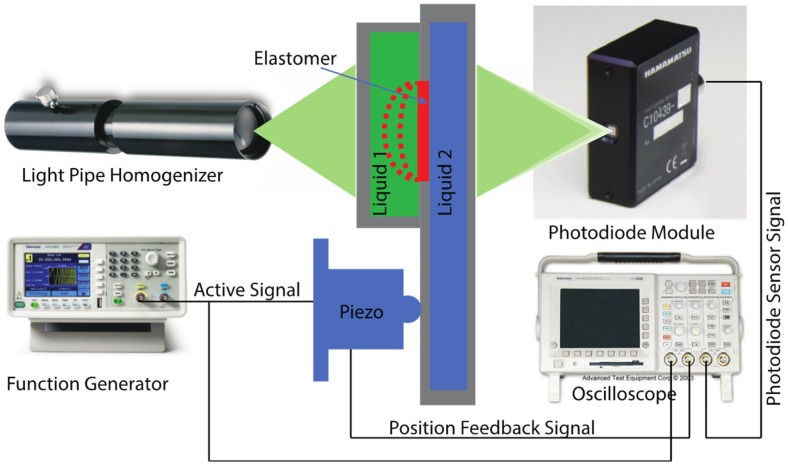
Schematic of the experimental setup used to measure the dynamic frequencies of the elastomer-based liquid-filled variable focus lens prototype.

A glass rod homogenizer (Model: RHO-13S-E2, Sigma Koki, Co., Ltd., St. Louis, MI, USA), which serves as a light guide, was used to illuminate the lens prototype. A photodiode module (Model: C10439-08, Hamamatsu Photonics K.K., Japan) was placed at the bottom of the lower liquid chamber to detect variations of the light luminance. When the focal length of the lens prototype changes, the density of the light that strikes on the photodiode module will change, which can be observed from variations of the output voltage. The variations of the output voltage of the photodiode module was observed from Channel 4 of the oscilloscope (Model: TDS 3034, Tektronix, Inc., USA). A function generator (Model: AFG1022, Tektronix, Inc., Beaverton, Oregon, OR, USA) was used to generate the active signal, which directs the motion of the piezo stack actuator (Model: P-841.60B, Physik Instrumente (PI) GmbH & Co., Karlsruhe, Germany). The motion of the piezo stack actuator was monitored on Channel 1 of the oscilloscope. The real-time position of the piezo stack actuator was observed on Channel 2 of the oscilloscope based on the feedback signal of the position sensor installed in the piezo stack actuator. Figure 6 shows the schematic of the experimental setup used to measure the dynamic frequencies of the elastomer-based liquid-filled variable focus lens prototype.

The lower chamber (blue chamber) was filled with pure water (density: ~0.997 g/cm^3^, refractive index: ~1.33), whereas the upper chamber (green chamber) was filled with polydimethylsiloxane (PDMS) (density: ~0.975 g/cm^3^, refractive index: ~1.40). The elastomer-taped plate was fixed on the connection hole as soon as the pure water was infused. Following this, the upper chamber was mounted on top of the elastomer-taped plate and the PDMS was infused.

A sinusoidal wave was used as the command signal to activate the piezo stack actuator and the frequency was gradually increased to confirm the vibration mode. When we used a low-frequency sinusoidal wave to generate the vibrations, we could observe the maximum amplitude of the feedback signal from the photodiode sensor. The amplitude range indicates the variations of the focal length of the lens prototype from minimum to maximum. As we increased the frequency of the sinusoidal wave, we observed that the lens prototype corresponds to the motion of the piezo stack actuator. However, when we used a high-frequency sinusoidal wave as the command signal, we observed that the amplitude of the feedback signal from the photodiode sensor significantly decreased due to vibrational damping.

## 4. Results and Discussion

**Figure 7:**
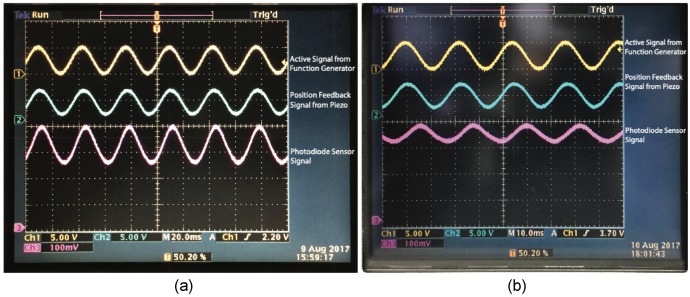
(**a**) Measured waves obtained for the lens prototype with an aperture size of 30 mm and the elastomer was stretched to 1.5 times its original length. The piezo stack actuator was moving at a frequency of 30 Hz with a full stroke of 90 μm. (**b**) Measured waves obtained for the lens prototype with an aperture size of 20 mm. The piezo stack actuator was moving at a frequency of 45 Hz with a full stroke of 90 μm. Similarly, the elastomer was stretched to 1.5 times its original length.

Figure 7 shows the measured waves displayed on the graphical user interface of the oscilloscope. Channel 1 (indicated by the yellow wave) refers to the sinusoidal wave with a frequency of 30 Hz generated from the function generator and transmitted to the piezo stack actuator. Channel 2 (indicated by the green wave) refers to the real-time position of the piezo stack actuator and it is evident that the deformation of the deformable plate corresponds to the displacement of the piezo stack actuator. Indeed, the light power changes with a change in the focal length and it can be observed from Channel 4 (indicated by the red wave) that the deformation of the elastomer surface corresponds to the applied signal. The threshold frequency of the vibration was 30 Hz when the aperture size of the lens prototype was 30 mm. When a high-frequency sinusoidal wave was applied, there was a pronounced decrease in the amplitude of the feedback signal from the photodiode module (as shown on Channel 4), indicating that the vibrations of the elastomer surface were damped. 

In another experiment, we stretched a piece of elastomer to 1.5 times its original length and the elastomer was taped onto a plate with an aperture size of 20 mm. Similarly, we applied a sinusoidal wave as the command signal of the piezo stack actuator and we gradually increased its frequency. We found that the vibrations of the elastomer surface were damped when the frequency of the applied signal was more than 45 Hz. The results confirm that the aperture size of the lens prototype has a significant effect on its natural frequency.

**Figure 8:**
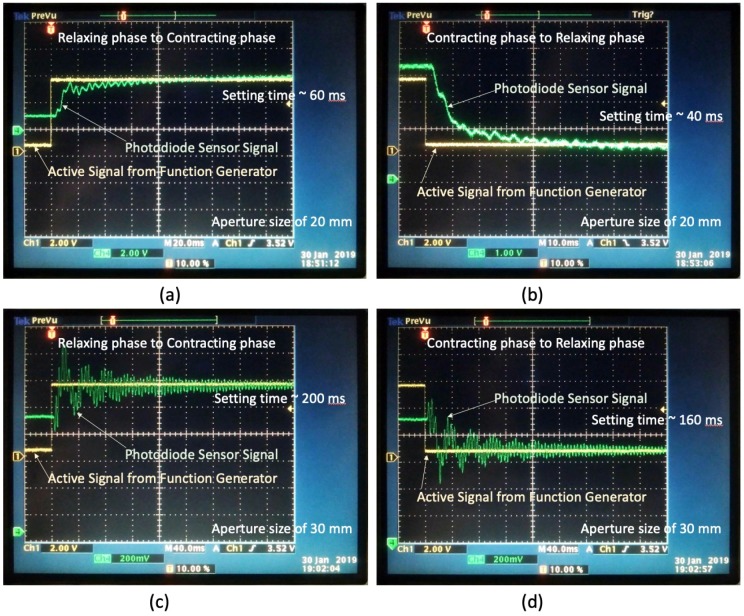
Measured responses of the elastomer-based liquid-filled variable focus lens prototype with an aperture size of (**a**, **b**) 20 mm and (**c**, **d**) 30 mm displayed on the graphical user interface of the oscilloscope. The settling times were 60 and 40 ms, respectively, when the lens prototype with an aperture size of 20 mm switched from the relaxing phase to contracting phase and vice versa. In contrast, the settling times were significantly higher for the lens prototype with an aperture size of 30 mm, with a value of 200 and 160 ms, respectively, when the lens prototype switched from the relaxing phase to the contracting phase and vice versa. The active signal (indicated by the yellow wave) was switched (**a**, **c**) from 0 V to 5 V and (**b**, **d**) from 5 V to 0 V.

Next, we performed experiments using a step signal (square wave) as the active signal. The response time for a square wave comprises the contracting time ($\tau_{contract}$) and relaxing time ($\tau_{relax}$). In this experiment, a square wave was applied to drive the lens prototype, where the active signal was switched between 0 V and 5 V. Thus, $\tau_{contract}$ and $\tau_{relax}$ are defined as the time taken for the lens prototype response to reach a steady-state condition when the applied signal is switched from 0 V to 5 V and from 5 V to 0 V, respectively. Figure 8 shows the measured responses of the lens prototype with an aperture size of 20 and 30 mm displayed on the graphical user interface of the oscilloscope. Channel 1 shows the square wave (indicated by the yellow wave) generated by the function generator whereas Channel 4 shows the variation of the focal length measured by the photodiode module (indicated by the green wave). 

It can be observed from the results that it takes a certain time for the response of the lens prototype to reach a steady-state condition. When the active signal was switched from 0 V to 5 V, the settling time required for the lens prototype with an aperture size of 20 mm to switch from the relaxing phase to the contracting phase ($\tau_{contract}$) was 60 ms. In contrast, when the active signal was switched from 5 V to 0 V, the settling time required of this lens prototype to switch from the contracting phase to the relaxing phase ($\tau_{relax}$) was reduced to 40 ms. We also used the same measurement procedure for the lens prototype with a larger aperture size (diameter: 30 mm) and we found that the settling time required for the lens prototype to switch from the relaxing phase to the contracting phase ($\tau_{contract}$) was 200 ms. Similarly, the settling time required for this lens prototype to switch from the contracting phase to the relaxing phase ($\tau_{relax}$) was slightly shorter, with a value of 160 ms. The results are in a good agreement with our previous discussion where the response time (settling time) of the elastomer-based liquid-filled variable focus lens will increase when the aperture size is increased. 

**Figure 9:**
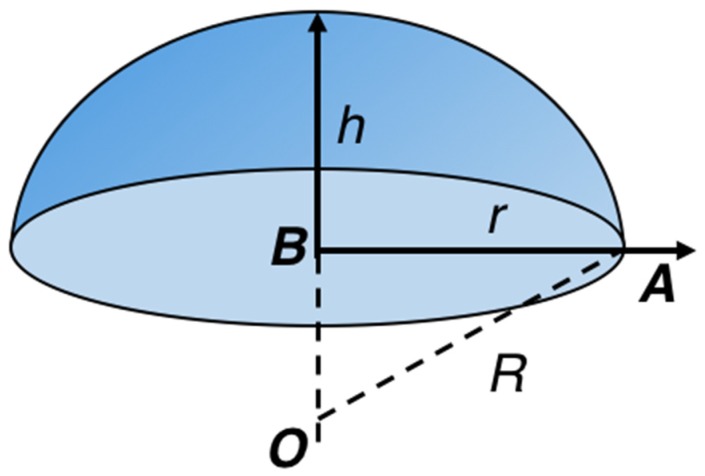
Schematic of the lens profile when the variable focus lens has a convex profile.

**Figure 10:**
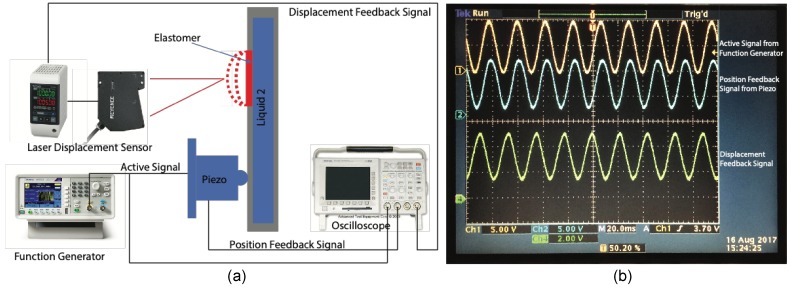
Schematic of the experimental setup (**a**) used to measure the displacement of the elastomer of the elastomer-based liquid-filled variable focus lens. The displacement at the center of the elastomer (**b**) (i.e., displacement at the center of the lens aperture) was measured by a high-speed laser displacement sensor (Model: LK-G400, Keyence Corporation, Osaka, Japan).

Figure 9 shows the tunable range of the elastomer-based liquid-filled variable focus lens, which was measured indirectly using the experimental setup in Figure 10. The displacement at the center of the elastomer was measured by a high-speed laser displacement sensor (Model: LK-G400, Keyence Corporation, Osaka, Japan). Because the elastomer and two liquids were transparent, it was difficult to measure the displacement of the elastomer membrane. Hence, we removed one of the liquid chambers and we affixed a small piece of paper at the center of the elastomer. With this modification, we were able to measure the displacement at the center of the elastomer when the laser beam struck the piece of paper. The laser displacement sensor was placed on top of the lens prototype. The maximum displacement was 2.2035 mm, indicating that the center of the elastomer was deflected from 0.0000 mm to 2.2035 mm. 

We assumed that the deformed elastomer surface was symmetric and uniform and the surface profile resembled a parabolic shape [14]. When the elastomer is not considerably deformed relative to its aperture size, the deformation can be approximated by a spherical shape [7]. Thus, the bending curvature can be calculated based on the displacement of the elastomer. As shown in Figure 9, in ABO, the curvature of the profile R, the aperture radius r, and length of OB (i.e., curvature *R_−_* displacement h) can be determined from the right-angled triangle. It shall be noted that $R^{2}={\left(R-h\right)}^{2}+r^{2}$.

When the aperture radius was 10 mm and the displacement was 2.2035 mm, the bending curvature was 23.793 mm. The power of the lens can be calculated from the following equation

(3) $$P=\frac{1}{f}=\frac{N_{1}-N_{2}}{R}.$$

N stands for the refractive index of the liquid. When the liquid pair consisted of pure water (refractive index: ~1.33) and silicone oil (refractive index: ~1.40), the tunable focal length ranged from 339.9 mm to infinity and the tunable power range was 0.00–2.94.

**Figure 11:**
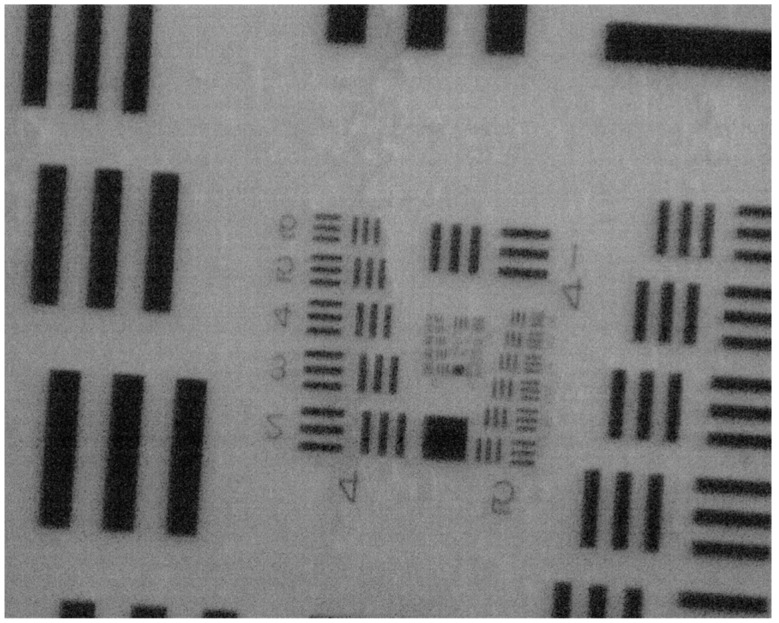
Image of the USAF 1951 resolution test chart captured when the focal length of the elastomer-based liquid-filled variable focus lens was 500.0 mm. The resolutions achieved were 64.00 lp/mm (Group 6, Element 1) and 50.80 lp/mm (Group 5, Element 5) in the vertical and horizontal directions, respectively.

The USAF 1951 resolution test chart was used as the target image and it was placed 39 mm away from the lens prototype. A camera (Basler acA1300-200uc, Basler AG, Ahrensburg, Germany) equipped with a microlens kit (Model: EO-39686 and EO-55359, Edmund Optics, Inc., Barrington, NJ, USA) were used to capture the image of the other size of the lens prototype. Figure 11 shows the recorded image when the focal length of the elastomer-based liquid-filled variable focus lens was 500.0 mm. We achieved a resolution of 64.00 lp/mm (Group 6, Element 1) and 50.80 lp/mm (Group 5, Element 5) in the vertical and horizontal directions, respectively.

## 5. Conclusions

In this work, we investigated the critical factors that affect the dynamic response of an elastomer-based liquid-filled variable focus lens. Based on theoretical analysis of a deformable circular disk (which is representative of a deformable surface), we found that the diameter, thickness, and stiffness of the circular disk are the critical factors that determine the first natural frequency of the variable focus lens. 

We fabricated the elastomer-based liquid-filled variable focus lens prototype, which consisted of a deformable plate and elastomer-taped plate with different aperture sizes (diameter: 20 and 30 mm). We used a high-speed piezo stack actuator as the pumping unit and we devised a mechanism to amplify the deformation of the deformable plate to the soft elastomer. The elastomer was stretched to 1.5 times its original length prior to fabrication to implant the in-plane pretension force device and increase the stiffness of the elastomer. 

When we applied a sinusoidal wave as the active signal on the deformable plate via the piezo stack actuator, we found that the lens prototype with an aperture size of 30 mm achieved a stable response at 30 Hz. In contrast, the lens prototype with an aperture size of 20 mm achieved a stable response at 45 Hz. When we applied a high-frequency sinusoidal wave on the deformable plate, we observed that the amplitude of the feedback signal from the photodiode module significantly decreased due to vibrational damping of the elastomer surface.

When we applied a square wave as the active signal, we found that the contracting time and relaxing time of the lens prototype with an aperture size of 30 mm were 200 and 160 ms, respectively. When we used a smaller aperture size (20 mm), the contracting time and relaxing time of the lens prototype were significantly reduced to 60 and 40 ms, respectively, indicating a marked improvement in the dynamic response of the elastomer-based liquid-filled variable focus lens. 

Because the elastomer-based liquid-filled variable focus lens can steadily vibrate to change its focal length at a frequency of more than 30 Hz, this lens can be potentially embedded in a high-speed projector to generate three-dimensional interactive scenes [20,21]. This lens can also be used to realize a high-speed shape from focus and all-in-focus imaging [22,23]. Even though the elastomer employed in our experiments can be easily fabricated, it suffers from low stiffness. Hence, the dynamic response of the elastomer-based liquid-filled variable focus lens can be significantly improved by employing an elastomer with a higher stiffness. In addition, the dimensions of the elastomer-based liquid-filled variable focus lens can be custom-tailored to fulfill the optical requirements for a particular application and the critical factors that affect the dynamic response identified in this work will be useful in the design of variable focus lens for optical systems.
